# S-Layer Ultrafiltration Membranes

**DOI:** 10.3390/membranes11040275

**Published:** 2021-04-08

**Authors:** Bernhard Schuster, Uwe B. Sleytr

**Affiliations:** Institute for Synthetic Bioarchitectures, Department of NanoBiotechnology, BOKU—University of Natural Resources and Life Sciences, Vienna, Muthgasse 11, 1190 Vienna, Austria

**Keywords:** S-layer protein, ultrafiltration membrane, biomimetic, nanotechnology, molecular sieving, immobilization of molecules, S-layer fusion-proteins, lipid membrane support

## Abstract

Monomolecular arrays of protein subunits forming surface layers (S-layers) are the most common outermost cell envelope components of prokaryotic organisms (bacteria and archaea). Since S-layers are periodic structures, they exhibit identical physicochemical properties for each constituent molecular unit down to the sub-nanometer level. Pores passing through S-layers show identical size and morphology and are in the range of ultrafiltration membranes. The functional groups on the surface and in the pores of the S-layer protein lattice are accessible for chemical modifications and for binding functional molecules in very precise fashion. S-layer ultrafiltration membranes (SUMs) can be produced by depositing S-layer fragments as a coherent (multi)layer on microfiltration membranes. After inter- and intramolecular crosslinking of the composite structure, the chemical and thermal resistance of these membranes was shown to be comparable to polyamide membranes. Chemical modification and/or specific binding of differently sized molecules allow the tuning of the surface properties and molecular sieving characteristics of SUMs. SUMs can be utilized as matrices for the controlled immobilization of functional biomolecules (e.g., ligands, enzymes, antibodies, and antigens) as required for many applications (e.g., biosensors, diagnostics, enzyme- and affinity-membranes). Finally, SUM represent unique supporting structures for stabilizing functional lipid membranes at meso- and macroscopic scale.

## 1. Introduction

Ultrafiltration (UF) is a membrane-based filtration, in which pressure or concentration gradients induce a separation through a semipermeable membrane. UF membranes processes are used in industry and research for purifying and concentrating suspended solids, macromolecules and colloidal particles, particularly protein solutions of 2 to 100 nm in diameter corresponding to a molecular weight of 10^3^ to 10^6^ Dalton (Da) [1,2,3]. UF membranes with a pore size ranging from 2 to 100 nm are defined by the specific molecular weight cut off (MWCO). The latter refers to the lowest molecular weight solute (in Da) in which 90% of the solute is retained by the membrane.

Beside the implementation of UF in research, UF membranes are an established separation technology utilized in many industrial process applications worldwide. UF membranes have the unique ability to purify, concentrate, and fractionate of a large range of macromolecules and proteins (e.g., milk proteins and enzymes, silt, plastics, oil, silica, nanoparticles, endotoxins, and viruses) via a physical membrane barrier determined by the MWCO. Thereby the UF membranes ensure a very consistent rejection and flux performance [2]. Industrial fields of application range from dairy, biotechnology and pharmaceutical use over food, beverage, and plant extracts to wastewater treatment [1,2,3].

Most UF membranes comprise of polymer materials like polysulfone, polypropylene, cellulose acetate, and polylactic acid [1,3] and have a surface porosity which is usually lower than 10%. They also show a size distribution of the pores varying by up to an order of magnitude [4]. Due to the presence of differently sized pores, the flux is strongly biased to the larger ones, with typically 50% of the solvent passing through 20 to 25% of the pores [4]. This leads to a heterogeneous flow pattern normal to the membrane surface. The heterogeneous porosity of UF membranes makes them susceptible to flux decline by loss of the larger pores [2,4] and thus, not well suited for the fractionation of close-sized macro-solutions.

**Figure 1:**
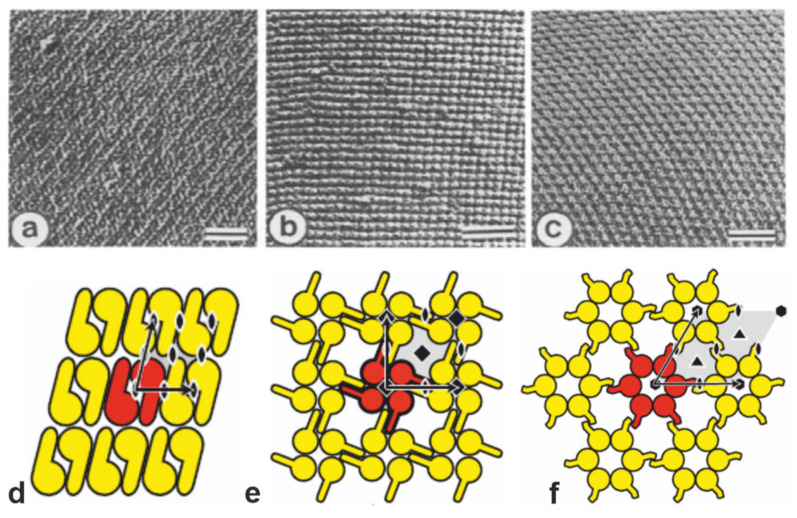
(**a**–**c**) Transmission electron micrographs of freeze-etched preparations of S-layer on intact cells showing three lattice types schematically determined in (**d**–**f**). (**a**,**d**) oblique (p2) lattice; (**b**,**e**) square (p4) lattice; (**c**,**f**) hexagonal (p6) lattice. The bars in a-c represent 50 nm. In d-f, the different S-layer lattice types, their base vectors, the unit cell (shaded in gray), and the corresponding symmetry axis are depicted. The proteins at one morphological unit are shown in red. (**a**–**c**: Adapted from [11]. Copyright © 1988 with permission from Elsevier Science Publishers B.V. Amsterdam, The Netherlands. **d**–**f**: With permission from Ref. [6] (CC BY-NC-ND 3.0)).

**Figure 2:**
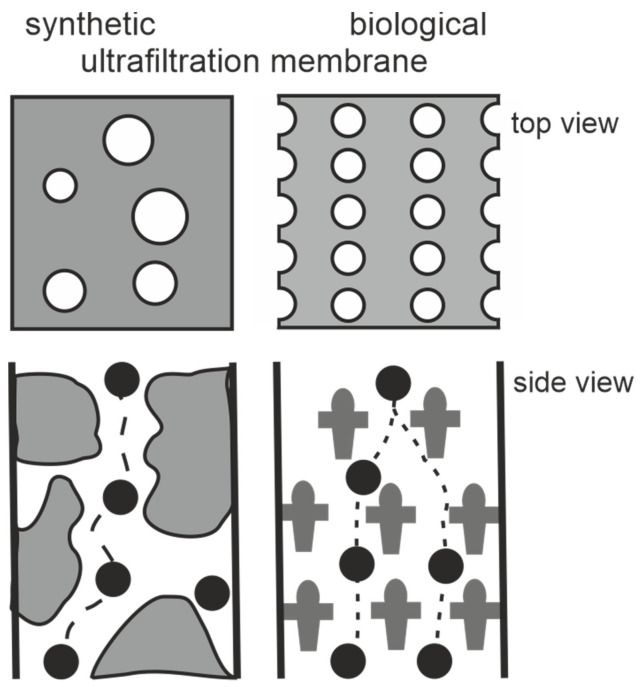
Schematic drawing of synthetic and S-layer ultrafiltration membrane. Left: The active ultrafiltration layers of most synthetic membranes show a porosity of up to 10%. Right: Crystalline S-layers reveal a porosity of up to approx. 70%. Although the active ultrafiltration layer is usually composed of several closed associated monolayers, the rejection characteristics is exclusively determined by the sieving properties of the individual S-layer protein monolayers. (Adapted from [8]. Copyright © 1986 with permission from Springer-Verlag).

In the present review we will introduce the highly porous crystalline monomolecular protein lattices, which represent the outermost surface layer (S-layer) in many bacteria and almost all archaea (Figure 1) as the MWCO-determining functional layer [5,6,7,8,9,10,11,12,13]. The S-layer ultrafiltration membrane (SUM) comprises of a microfiltration membrane onto which S-layer-carrying cell wall fragments or S-layer protein (SLP) self-assembly products are deposited in a pressure-dependent manner [6,8,9,14,15,16,17,18,19]. Whereas the active ultrafiltration layers of most synthetic membranes show a porosity of up to 10%, the crystalline S-layers reveal a porosity of up to 70% (Figure 2). The preformed SUM can, if desired, be chemically crosslinked to enhance the mechanical and thermal robustness. In the following we will report on the intrinsic features of bacterial SLPs, their molecular sieve property and how SUM are manufactured and modified to show optimal performance in filtration processes. Moreover, SUMs may also be utilized as binding matrices for the formation of complex composite structures for various biotechnological and pharmaceutical applications.

## 2. Ultrastructure and Self-Assembly of S-Layer Proteins

Monomolecular arrays of protein or glycoprotein subunits forming S-layers are one of the most observed prokaryotic cell envelope components [6]. The isoporous lattices covering the entire cell surface provide organisms with various selection advantages including functioning as protective coats, molecular sieves, and ion traps, as structures involved in surface recognition and cell adhesion, and as antifouling layers [6]. S-layers completely cover the cell surface during all stages of cell growth and division [20,21,22,23,24,25,26]. The location and ultrastructure of S-layers of a great number of *Bacteria* and *Archaea* have initially been studied by transmission electron microscopy (TEM) (Figure 1a–c) [21,27,28,29,30,31,32,33] and more recently by atomic force microscopy (AFM) [34,35,36,37,38,39]. It turned out that S-layers exhibit either oblique, square, or hexagonal lattice symmetry, where the number of SLPs per morphological unit is either one or two (p1, p2; oblique), four (p4; square), or three or six (p3, p6; hexagonal) (Figure 1d,e). The unit cell dimension ranges from 3 to 30 nm for bacterial and archaeal S-layer lattices. The morphology of the S-layer lattice is asymmetric with commonly a smooth outer face and a more corrugated inner face (with respect to their orientation at the cell). Finally, bacterial S-layer lattices are highly porous protein mesh works (30 to 70% porosity) with pores of uniform morphology and size (between 2 to 8 nm) and a thickness between 5 and 20 nm [6,20,40,41,42,43,44].

Generally, S-layers are isolated from cell wall fragments, which were obtained by breaking up the cells and removing their content, including the cytoplasmic membrane. Most often, hydrogen-bond breaking agents like guanidine hydrochloride or urea are used to disintegrate and solubilize the SLPs. For a detailed compilation of protocols, see reference [45].

The capability of isolated SLPs to assemble into two-dimensional arrays in vivo and in vitro is one of their key properties exploited in basic and application-oriented research. It occurs upon dialysis of the disrupting agents [6,45,46,47]. The formation of the monomolecular self-assembled protein lattices is only determined by the amino acid sequence of the polypeptide chains, and consequently the tertiary structure of the SLP species [48].

The assembly process in solution shows multiphasic kinetics with a rapid initial and a slow consecutive phase [49]. The rapid phase may be attributed to the formation of oligomeric precursors. The latter fuse in the consecutive phase and reassemble into the finally formed S-layer self-assembly products [6,46,47,49,50,51]. SLPs are non-covalently linked to each other and, in the case of their adhesion to supporting structures (e.g., microfiltration membrane or polymeric solid surfaces) combinations of weak bonds (hydrophobic bonds, ionic bonds involving divalent cations or direct interaction of polar groups, and hydrogen bonds) are responsible for the structural integrity as well [6]. Once formed, SLPs were never observed to leave the lattice, and thus, it was concluded that lattice growth is irreversible. The reason for the latter may be that after the addition of the “last” protein monomer to the (incomplete) morphological unit, this monomer is locked into place and now has a low probability of leaving [34,52].

## 3. S-Layers as Molecular Sieves

To determine the size of pores in S-layer lattices of different *Bacillaceae*, permeability studies were performed. Native and glutaraldehyde-treated S-layer containers were prepared that resembled the shape of bacterial cell envelopes [53]. The solutions selected for the molecular sieving measurements were sugars, proteins, and dextrans of increasing molecular weights. It was clearly demonstrated that the S-layer lattices are isoporous molecular sieves that allow free passage for molecules with a molecular weight of up to 30.000 Da and showed sharp exclusion limits between molecular weights of 30.000 and 45.000 Da [8,11,53]. This finding suggests a limiting pore diameter in the range of 3–4.5 nm, which resembles the pore dimensions determined by high-resolution TEM and AFM [27,54]. This information on the structure and function of different S-layers of *Bacillaceae* makes it unlikely that their S-layers have the potential to function as an effective barrier against lysogenic enzymes [53]. Most important, a great variety of permeability studies on S-layers from *Bacillaceae* demonstrated that the surface and pore areas of the protein meshwork have a very low tendency for unspecific adsorption of (macro)molecules [10,12,18,55,56,57,58].

## 4. S-Layer Ultrafiltration Membrane (SUM)

**Figure 3:**
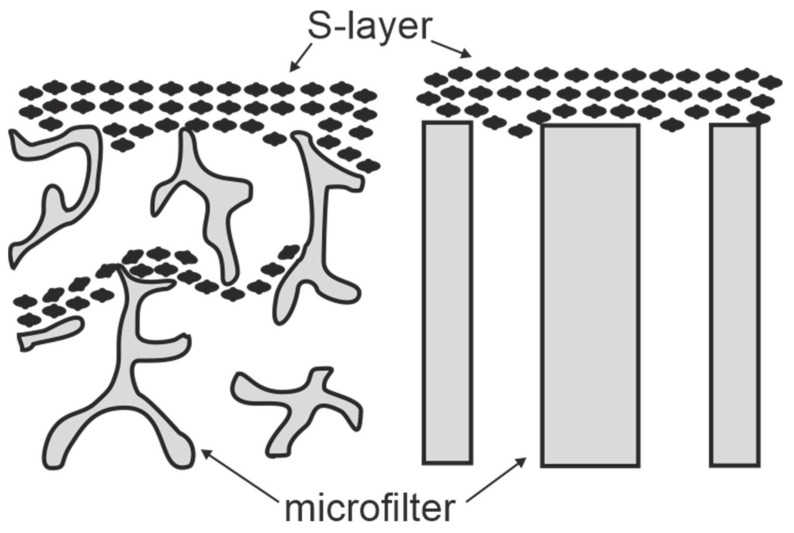
Schematic drawing of S-layer ultrafiltration membranes. Left: S-layer fragments are deposited on the surface and in the substructure of open-celled foam-like microfiltration membranes. Right: S-layer fragments are attached to the surface of radiation-track membranes. (Adapted from [8]. Copyright © 1986 with permission from Springer-Verlag).

SUMs are made by depositing either sheet like S-layer self-assembly products (see Section 2) or S-layer carrying cell wall fragments (approx. 0.5 to 1.5 µm size) on commercial microfiltration membranes with a spongy structure or radiation-track type membranes having pore sizes of 0.05 to 0.1 µm in a pressure dependent procedure (Figure 3) [10,11,59,60]. S-layer carrying cell wall fragments were prepared from whole cells by extracting the cytoplasmic membrane with Triton X-100 under conditions, which preserved the integrity of the S layer and the peptidoglycan-containing layer [53]. Like tiling a roof, this procedure generates a coherent coating, composed of multilayers of S-layer fragments arranged in random orientation. Interestingly, the pores of the constituent (monomolecular) S-layer lattice solely determined the MWCO of these composite multilayered structures.

**Figure 4:**
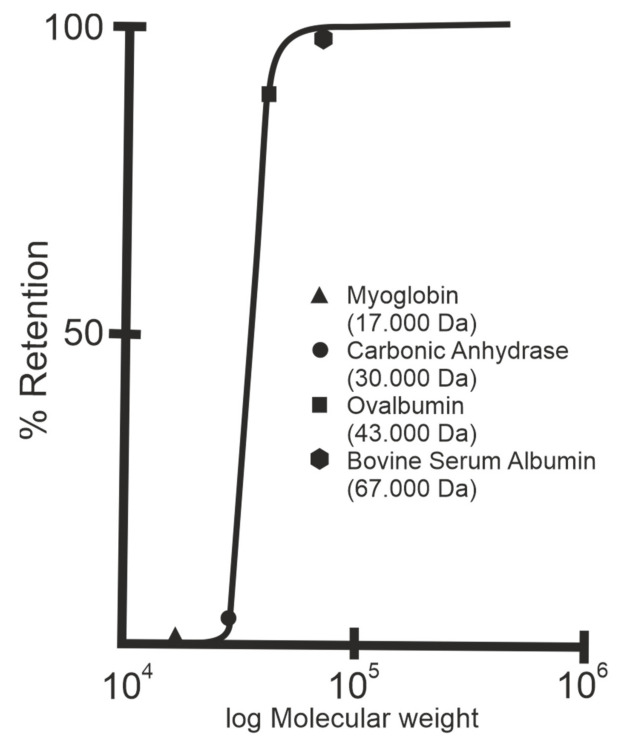
Rejection curve of S-layer ultrafiltration membranes. The isoporous S-layer fragments attached to a supporting microfiltration membrane form the active ultrafiltration layer responsible for the sieving properties. Note the steep increase between molecular weights 30.000 and 45.000 Da for S-layer fragments of *Geobacillus stearothermophilus*. (Adapted from [8]. Copyright © 1986 with permission from Springer-Verlag).

After deposition, the SLP lattices are inter- and intramolecular crosslinked with glutaraldehyde and Schiff bases are reduced with sodium borohydride [10]. The nominal MWCO of SUMs from S-layers of mesophilic and thermophilic *Bacillaceae* revealed no significant difference and was in the range of 30.000 to 40.000 Da (Figure 4) [10,12,53]. As estimated by TEM and AFM, the porosity of the SLP lattice lies between ca. 30 and 70% and is therefore significantly higher than that determined for polymeric UF membranes with a maximum of 10%.

Native S-layers were found to be zwitterionic, thus preventing nonspecific adsorption of charged macromolecules, which would lead to pore blocking (fouling) [61]. After crosslinking the S-layer protein lattice with glutaraldehyde, which involves loss of positively-charged amino groups, SUMs revealed a net negative charge on the surface and inside the pores [10,17,19,62]. SUMs possessing a high density of free carboxylic acid groups did not adsorb negatively charged macromolecules but strongly interacted with proteins exhibiting a positive net charge [14,41,56]. In ultrafiltration processes, protein adsorption, which is considered as the first step in membrane fouling is expressed in terms of flux losses for particle free water after protein filtration [63,64]. By using SUMs composed of two-dimensional crystalline protein lattices with defined pore sizes and defined surface charge, it was possible for the first time to determine correlations between the pore size and the net charge of the active filtration layer, the molecular characteristics (dimensions, net charge) of adsorbed protein molecules and the flux losses caused by adsorption [19,62,65]. The flux of SUMs produced with S-layers from *Geobacillus* strains ranged from 150 to 250 L m−2 h−1 when measured at 0.2 MPa with water [12]. Although the initial flux of SUMS correlated with the number of S-layer fragments deposited on the microfiltration membrane, the dimension of the active filtration layer did not influence the MWCO [10,59,60]. As S-layers are periodic structures, they exhibit repetitive identical physicochemical properties down to the sub-nanometer scale not only on the surface but also in the pore area. Thus, surface properties and molecular sieving as well as antifouling characteristics of SUMs could be tuned by chemical modifications involving activation of carboxylic acid groups with carbodiimides and subsequently converting them with differently sized and charged nucleophiles [41,42,62,66]. Thereby, SUMs can be manufactured with different net charge, hydrophilic or hydrophobic surface properties and separation characteristics [62]. A correlation was observed between the molecular size of attached nucleophiles and the shift of the sharp rejection curve to the lower molecular weight range [17,62]. This confirmed that a pore size reduction had occurred due to an aperture like enlargement of modified carboxylic acid groups exposed in precise position and orientation on protein domains inside the pores. It was also demonstrated that both, the net charge of the S-layer lattice and that of the protein molecules used in filtration experiments determine the solute rejection characteristics of SUMs [42,62,67]. Thus, reams of chemical and/or genetically induced modifications allow the adaptation of SUMs to very specific process requirements. In comparison with conventional ultrafiltration membranes produced by amorphous polymers, SUMs exhibited an extremely low unspecific protein adsorption (membrane fouling) in buffer solutions. Concerning the chemical stability, SUMs composed of inter- and intramolecular crosslinked S-layer lattices were resistant towards many organic solvents (ketones, alcohols, chlorinated hydrocarbons, aromatic compounds), chaotropic agents, acidic and alkaline pH conditions (pH 1–13 at 20 °C for 160 h), and shear forces. These studies demonstrated that SUMs are equivalent in their chemical resistance to polyamide membranes [10,14,18,66,68].

## 5. S-Layers as Matrix for the Immobilization of Functional Macromolecules

**Figure 5:**
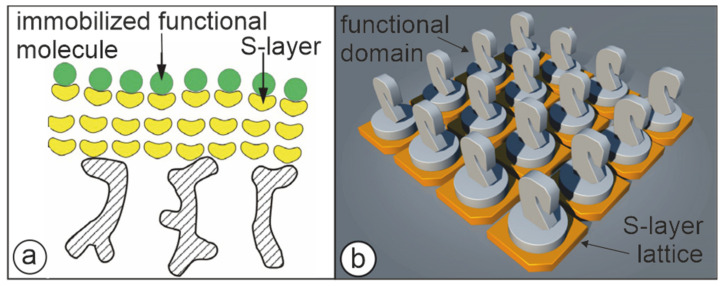
Cartoons illustrating the two major approaches to present functional molecules or domains on S-layer lattices. (**a**) S-layer ultrafiltration membrane comprising of S-layer proteins with covalently attached functional molecules. (Adapted from [72]. Copyright © 2011 with permission from Springer-Verlag). (**b**) Self-assembled S-layer fusion proteins carrying functional domains (represented as knights) in precise position and orientation. (With permission from Ref. [6] (CC BY-NC-ND 3.0)).

Immobilization procedures for macromolecules can be classified into crosslinking, entrapping and carrier-binding, which is further subdivided into physical adsorption, ionic binding and covalent binding [69,70]. Covalent binding is considered the preferred method since the forces between the macromolecules and the carrier are strong and leakage of the immobilized molecules is prevented under disrupting conditions such as high salt concentrations or major pH shifts. The commonly used carriers for immobilizing macromolecules are particles or membranes with spongy structures made of various polymers. Despite their different chemical composition, they reveal an amorphous structure, which imply a random distribution and orientation of the functional groups. With SLP lattice immobilization matrices with a characteristic topography and a defined arrangement and orientation of functional groups became available [18,20,42,44,71,72]. These characteristic features allow for the first time a reproducible and geometrically well-defined binding of macromolecules with a precision down to the sub-nanometer scale (Figure 5). Because of their high stability particularly regarding to shear forces, SUMs were additionally utilized as a matrix for immobilizing functional molecules [20,42,68,71,72,73,74,75]. For immobilizing functional molecules, carboxylic acid groups on the surface of the crosslinked S-layer matrix were activated with water-soluble carbodiimide. The thus activated groups can subsequently react with free amino groups of the functional macromolecules leading to stable peptide bonds between the S-layer matrix and the immobilized protein such as enzymes, antibodies, antigens or ligands (e.g., streptavidin, protein A and G) (Figure 5a) [15,76]. An alternative method involved the modification of the S-layer lattices with 2-iminothiolane, which introduced sulfhydryl groups that could be exploited for further modification reactions or for covalent binding of sulfhydryl group-containing molecules [42,77].

From the amount and size of macromolecules that could be bound to individual S-layer lattices with known lattice constants and defined molecular mass of the S-layer constituent subunits, it was derived that most macromolecules formed a dense monolayer on the surface of S-layer lattices [42,78,79,80,81]. The binding capacity of 10 nm-sized ferritin could be visualized by TEM [76]. Large enzymes (e.g., invertase, mutarotase, glucose oxidase (GOx), maltase, xanthine oxidase, glucuronidase, β-galactosidase, alcohol oxidase, and laccase) were directly bound to the S-layer matrix (Figure 5a) [17,42,56,81,82,83,84,85,86,87,88,89] whereas smaller enzymes were preferentially immobilized involving spacer molecules to retain their enzymatic activities [90]. Although studies on the density of bound molecules provided evidence for a preferential orientation on the S-layer matrix, an unsurpassed precision in orientation and distribution of bound molecules was obtained by the assembly of fusion proteins comprising an SLP and a functional protein [6,91]. This genetic engineering approach enabled the production of sheet like S-layer lattices where the functional molecules are arranged in defined position and orientation like a chessman on a chessboard (Figure 5b) [6,73,91,92]. Both methods, direct immobilization of functional molecules on S-layer surfaces and the utilization of S-layer fusion proteins were exploited for a great variety of applications [6]. For example, universal bio-specific matrices for immunoassays and dipsticks could be generated by immobilizing monolayers of streptavidin, protein A or protein G [93]. Other SUM-based dipsticks were produced for quantification of tissue type plasminogen activator (t-PA), interleukin 8, type I allergies [80,94,95] and prion diagnosis [96]. Due to the dense packing of the immobilized functional molecules, no unspecific adsorption of molecules occurred even when dipsticks were used in complex media like blood serum.

**Figure 6:**
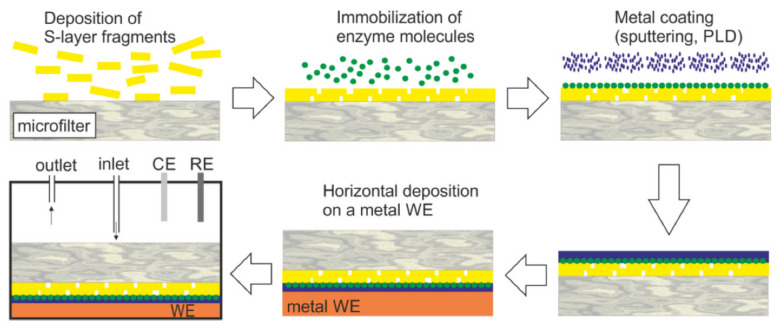
Generalized scheme of the construction of an S-layer ultrafiltration membrane (SUM)-based biosensor (not drawn to scale). S-layer carrying fragments or self-assembly products are deposited on a commercially available microfilter in a pressure-dependent process. The enzyme is deposited on the S-layer surface and chemically linked to the S-layer protein. The immobilized enzyme is contacted by a thin metal layer (PLD: Pulse-laser-deposition). Finally, this composite structure is deposited with the metal layer side on the working electrode (WE) and mounted in a flow cell (RE: Reference electrode, CE: Counter electrode). The analyte is pumped into the flow cell. After passage across the modified SUM, the analyte reacts with the enzyme. This reaction can be detected by amperometry. (With permission from Ref. [98] (CC BY 4.0)).

SUMS were also used as supporting matrix for biologically active components to produce amperometric biosensors [82,83,87,97,98]. In Figure 6, a generalized scheme of the construction of an SUM-based biosensor is shown. The single and multienzyme amperometric biosensors based on SUM technology were successfully constructed to detect glucose using GOx [82], ethanol using alcohol oxidase [87], cholesterol using cholesterol esterase and cholesterol oxidase [87], xanthine using xanthine oxidase [87], maltose using maltase and GOx [87], and a multienzyme biosensor using invertase, mutarotase, and GOx for the detection of sucrose [83]. A thin metal coating was generated either by sputtering or pulse-laser deposition in order to achieve a better electron transfer between the immobilized enzymes and the working electrode. The glucose and sucrose sensor showed a linear range of up to 12 mM and 30 mM and a stability of 48 h and 36 h, respectively [82,83,98]. The fields of application of the above described SUM-based biosensors are blood analysis, microbiology, and food technology [87].

## 6. SUM as Supporting Scaffold for Functional Lipid Membranes

**Figure 7:**
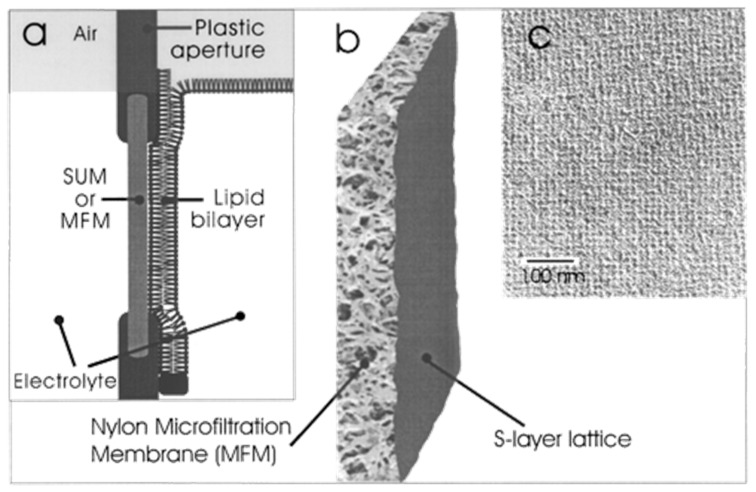
S-layer ultrafiltration membrane (SUM). (**a**) Schematic drawing of an SUM-supported phospholipid bilayer (not drawn to scale). The lipid membrane was generated on the S-layer face of the SUM. (**b**) S-layer fragments were deposited with pressure on a commercially available Nylon microfiltration membrane. (**c**) Transmission electron micrograph of a platinum/carbon replica of a freeze-dried SUM composed of the S-layer protein *Lysinibacillus sphericus* CCM 2120 showing the square S-layer lattice. (Adapted from [100]. Copyright © 2001 with permission from American Chemical Society).

A widely used technique to generate bilayer lipid membranes (BLM) is to fold the lipid membrane consecutively out of two lipid monolayers over a small plastic aperture [99]. These so-called folded BLMs are easy to generate and are very properly suited to reconstitute even bulky membrane proteins. However, one crucial drawback is the restricted longevity, that means that the lipid membrane is susceptible to mechanical vibration and tends to brake after minutes to few hours. The idea came up to put on one side of the aperture a filtration membrane in order to reduce possible mechanical stress to the BLM [100,101,102]. On the one hand side, the bare microfiltration membrane (MFM), which is used in the SUM production and on the other hand, the SUM has been used for this purpose (Figure 7). This strategy was successful as determined by applying a voltage ramp with a slope of 10 mV/s and a maximal voltage of 500 mV [100]. Whereas the folded BLM comprising of 1,2-diphytanoyl-*sn*-glycero-3-phosphocholine (DPhPC) ruptured at the application of the first voltage ramp at a voltage of 220 mV and the MFM-supported BLM at the second ramp at a voltage of 210 mV, the SUM-supported BLM survived three voltage ramps up to 500 mV. Moreover, the latter SUM-supported membrane revealed the by far the longest lifetime of 17 h compared to the other two BLMs [102].

The above given data on the stability are for BLMs comprising of DPhPC. However, also the main phospholipid of *Thermoplasma acidophilum* (MPL), a bipolar tetraether lipid was used for lipid membrane formation [101,102]. Whereas DPhPC membranes were generated on the SUM in a two-step approach, only one-step was sufficient to generate the membranes containing MPL. Interestingly, it was also possible to form stable membranes out of mixtures of DPhPC and MPL by applying the one-step approach. A MPL monolayer shows the same structural properties than the DPhPC phospholipid bilayer [101,102]. Obviously, DPhPC and MPL are able to intermix with each other in order to form the typical bipolar structure of lipid membranes.

Whereas composite SUM-supported DPhPC bilayers and SUM-supported MPL monolayers were found to be highly isolating structures with a lifetime of up to 17 h and 18 h, respectively [100,101,102], BLMs on plain microfiltration membranes revealed a lifetime of approximately 3 h. The lifetime increased significantly to about 1 day by formation of an S-layer–lipid membrane–S-layer sandwich-like structure, i.e., an additional monomolecular S-layer protein lattice recrystallized on the lipid-faced side of the SUM-supported MPL membrane [101]. An even further increase in the stability of this composite supramolecular structure can be expected upon crosslinking of those lipid head groups involved in direct contact with the S-layer proteins. Hence, the nanopatterned anchoring of the membrane is a promising strategy for generating stable and fluid lipid membranes [103,104].

Another very important feature of the SUM-supported lipid membranes is beside their elevated longevity the possibility to incorporate membrane-active peptides and the transmembrane proteins like, e.g., alpha-hemolysin (αHL). Functional αHL pores can be reconstituted in SUM-supported DPhPC membranes and the unitary conductance of the αHL pore was determined from current steps as pores assembled and inserted or by the closing of single pores. The specific conductance of a single αHL pore determined at +40 mV was by approx. 10% reduced for SUM-supported compared to free-standing folded DPhPC membranes. From this finding, one can conclude that the αHL “sees” the underlying SUM to a certain extent but the ions flux through the αHL pore is largely retained [100]. The pore-forming peptide gramicidin was incorporated in SUM-supported lipid membranes comprising DPhPC, MPL, and of mixtures of DPhPC and MPL [101]. It was demonstrated, that even single gramicidin pore measurements could be performed in all SUM-supported membranes. Thus, DPhPC and MPL as well form electrically isolating membranes on the SUM, which provide a suitable thickness and fluidity for the functional insertion of gramicidin pores [101]. Moreover, also the membrane-active peptides valinomycin and alamethicin were successfully reconstituted in SUM-supported lipid membranes as determined by electrochemical impedance spectroscopy [102].

## 7. Conclusions and Outlook

**Figure 8:**
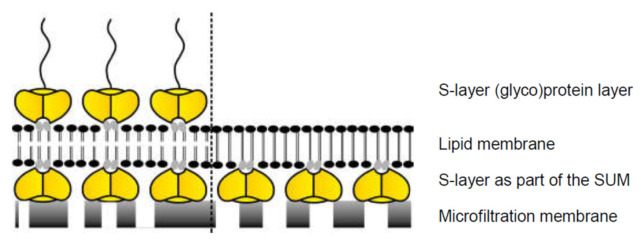
Schematic drawing of lipid membranes on S-layer (yellow)-covered microfilters. Some head groups of the lipid molecules within the membrane (gray) interact electrostatically with certain domains on the S-layer lattice. A further (glyco)protein S-layer lattice can be recrystallized on the outer leaflet of the lipid membrane (left). In analogy, some head groups of the lipid molecules within the membrane (gray) interact electrostatically with certain domains of the S-layer proteins. The lipid molecules on the left side depict schematically phospholipids, whereas the lipid molecules on the right side indicate tetraether lipids. (With permission from Ref. [6] (CC BY-NC-ND 3.0)).

As originally, proposed by Heckmann and coworkers [105,106], hyperfiltration membranes may be produced by depositing and crosslinking mono- or double layers of surfactants (e.g., tetraether lipids) on plane ultrafiltration membranes. Considering the smooth surface and high porosity of SUMs such potential application should be remembered. This supramolecular construction principle would resemble archaeal cell envelopes composed of a plasma membrane and a closely associated S-layer lattice (Figure 8). Moreover, on the surface of the hyperfiltration membrane additional S-layer fragments may be deposited leading to an “S-layer-sandwiched hyperfiltration membrane”. Most efficient antifouling properties would be achieved when the outer coating would be composed of glycosylated S-layers (Figure 8) [107]. After intra- and intermolecular crosslinking such an outer S-layer coating will provide a very efficient protective coat for the delicate monolayer-hyperfiltration membrane. 

Although the direct binding of functional molecules onto SUMs will be for many scopes sufficient, the use of S-layer fusion proteins will improve and simplify manufacturing processes. To date, a variety of S-layer proteins assembling with different lattice constants into oblique (p1) and square (p4) lattices were selected as fusion partners for the construction of chimeric S-layer proteins [91]. It was confirmed that the self-assembly properties conferred by the S-layer moiety as well as the functionalities of the fused peptide sequences were retained in all fusion proteins tested [6]. Most remarkable, the functional S-layer proteins (e.g., hypervariable region of heavy chain camel antibodies, prostate specific antigen, two copies of the Fc-binding-Z-domain, a synthetic analogue of the B-domain of protein A, the major birch pollen allergen-Bet v1, peptide mimotopes, different fluorescent proteins, core streptavidin, or monomeric and multimeric enzymes from extremophiles) could be successfully reassembled into monomolecular arrays [6,72,91,108]. Moreover, the functional moieties maintained their functionality much better when part of a constituent S-layer subunit in comparison when being covalently bound to SUMs [6]. In other words, it can be predicted that future SUM technologies will be based on the deposition of plane assemblies of chimeric S-layer proteins on microfiltration membranes followed by an inter- and intramolecular crosslinking step. Crosslinking with dimethyl pimelimidate dihydrochloride was demonstrated as suitable method for maintaining the functionality of the chimeric proteins [72,109].

The information accumulated on the structure, chemistry, genetics, assembly, and function of S-layers has led to reams of applications in nanotechnology and biomimetics [6]. Many areas of applied S-layer research will be promoted in the future by the construction of S-layer fusion proteins composed of the S-layer assembly domain and a functional moiety [91]. Such functionalized protein lattices possess a considerable application potential as patterning element for generating more complex, supramolecular assemblies involving all relevant molecules such as proteins, lipids, glycans, and nucleic acids [6].

One of the most striking features of native S-layers are their excellent antifouling properties. S-layers on cells, even harvested from most complex environments, were never masked by absorbed molecules as confirmed by electron microscopy [20,21,22]. This so-called “Nano-Lotus-effect” [6] is partially explained by the fact that they have a charge neutral zwitterionic surface preventing nonspecific binding of molecules and most important pore blocking [20]. Nature has optimized during billions of years of evolution a nanoscopic architecture, which provides self-cleaning properties. Thus, SLP lattices fulfills most relevant requirements for a molecular sieve in the ultrafiltration range. Another feature of S-layer lattices, which might significantly contribute to their functionality are their repetitive topographical characteristics and the optional attachment of carbohydrate residues on the surface [107]. In this context. it should be considered that the self-cleaning capability of biological surfaces relies not only on the wettability but rather on the structure of the water molecules near the substrate [107,110]. Further studies will show if S-layer lattices possess this “Nano-Lotus-effect”, which might be even copied by advanced polymer chemistry in combination with a specific nanopatterning of the surface.

The present review underlines that the application potential of SUMs is manifold, as they constitute a unique filtration layer with sharp MWCO combined with self-cleaning properties. The porosity of SUMs is with up to 70% remarkably high, which is of advantage for filtration application and make them ideal as supporting structures for stabilizing functional lipid membranes at meso- and macroscopic scale. SUMs can be utilized as matrices for the controlled immobilization of functional biomolecules (e.g., ligands, enzymes, antibodies, and antigens) as required for many applications including enzyme- and bio-specific affinity matrices for immune assays and diagnostics. Moreover, very sensitive and robust biosensors have been developed for the application in microbiology, biotechnology, food technology, pharmacy, and blood analysis.

Finally, it should be stressed that until now all studies with SUM’s were performed with S-layer-(glyco)protein lattices derived from Bacteria. Since some Archaea, possessing S-layers as exclusive cell wall component dwell under extreme environmental conditions (e.g., 120 °C, pH 0, concentrated salt solutions) it will be interesting to exploit their S-layers. Apparently, some are very resistant to disintegration into their constituent glycoprotein subunits, (e.g., by boiling in SDS) indicating the possible presence of covalent inter-subunit bonds [26,32,111]. SUMs made of S-layers from extremophiles should enable to produce membranes with very little leakage and excellent biocompatibility as required for blood purification and fractionation (e.g., aphaeresis), implants, and biosensors development [97,98].

Electron microscopical data provide strong evidence that pores in S-layers lattices from Archaea are larger than those observed in Bacteria. Consequently, such membranes should reveal higher MWCOs and thus, extend their application potential into a higher molecular weight range. Accumulated data provide evidence that it would be possible to scaling up SUM production using both S-layers from Bacteria and Archaea. Moreover, continuous SUM assembly lines may be developed for manufacturing flat or hollow fiber polymeric ultrafiltration membranes. The production of SLPs as a biological component at low-cost and in a reproducible manner is still a challenge in particular for Archaea as their cultivation and SLP isolation has to occur under harsh conditions. This can also be seen by the fact that so far the Bio-Technology Readiness Level of archaeal SLPs is in the proof-of-principle phase and has thus to be pushed up significantly in order to be utilized in commercial applications [112].

## 8. Patents

Sleytr, U. B., Sara, M. (1988): Structure with membrane having continuous pores. US Patent Number 4,752,395 (21 June 1988).

Sleytr, U. B., Sara, M. (1989): Use of structure with membrane having continuous pores. US Patent Number 4,849,109 (18 July 1989).

## Data Availability

Publicly available datasets were analyzed in this review. This data can be found in the respective cited references.
